# Supplementary material for: Enzyme-mimetic self-catalyzed polymerization of polypeptide helices
Source: Nat Commun. 2019 Nov 29;10:5470. doi: 10.1038/s41467-019-13502-w (PMC6884638; doi:10.1038/s41467-019-13502-w)
Supplement: Supplementary file 1 — Supplementary Information [file 41467_2019_13502_MOESM1_ESM.pdf]

Supplementary Information

Enzyme-mimetic self-catalyzed polymerization of polypeptide helices

Song et al.

Supplementary Methods

Materials

All chemicals were purchased from MilliporeSigma (St. Louis, MO, USA) unless otherwise specified. Amino acids were purchased from Chem-Impex International Inc. (Wood Dale, IL, USA). Deuterated solvents were purchased from Cambridge Isotope Laboratories, Inc. (Tewksbury, MA, USA). Anhydrous *N*,*N*-dimethylformamide (DMF) and deuterated DMF (DMF-*d*7) were treated with polymer-bound isocyanates (MilliporeSigma, St. Louis, MO, USA) to remove any amine residues. Anhydrous dichloromethane (DCM) and deuterated DCM (CD_2Cl_2) were stored over 3 Å molecular sieves in a freezer. γ-Benzyl-L-glutamate *N*-carboxyanhydride (BLG-NCA)[1], γ-benzyl-D-glutamate *N*-carboxyanhydride (BDG-NCA)[1], γ-ethyl-L-glutamate *N*-carboxyanhydride (ELG-NCA)[1], and N^{ε}-2-(2-(2-methoxyethoxy)ethoxy)acetyl-L-lysine NCA (EG_2-Lys-NCA)[2], sarcosine *N*-carboxyanhydride[3] were synthesized following literature procedures.

Instrumentation

Proton nuclear magnetic resonance (NMR) spectra were recorded on a Varian U500 or VNS750NB spectrometer in the NMR laboratory, University of Illinois. Chemical shifts (*δ*) were reported in ppm and referenced to the residual protons in the deuterated solvents. MestReNova software (version 8.1.1, Mestrelab Research, Escondido, CA, USA) was used for all NMR analysis. Gel permeation chromatography (GPC) experiments were performed on a system equipped with an isocratic pump (1260 Infinity II, Agilent, Santa Clara, CA, USA), a multi-angle static light scattering (MALS) detector (DAWN HELEOS-II, Wyatt Technology, Santa Barbara, CA, USA), and a differential refractometer (DRI) detector (Optilab T-rEX, Wyatt Technology, Santa Barbara, CA, USA). The detection wavelength of HELEOS was set at 658 nm. Separations were performed using serially connected size exclusion columns (three PLgel MIXED-B columns, 10 μm, 7.5 × 300 mm, Agilent, Santa Clara, CA, USA) at 40 ℃ using DMF containing 0.1 mol/L LiBr as the mobile phase. The MALS detector was calibrated using pure toluene and can be used for the determination of the absolute molecular weights (MWs). The MWs of polymers were determined based on the *d*n/*d*c value of each polymer sample calculated offline by using the internal calibration system processed by the ASTRA 7 software (version 7.1.3.15, Wyatt Technology, Santa Barbara, CA, USA). Fourier transform infrared (FTIR) spectra were

recorded on a Perkin Elmer 100 serial FTIR spectrophotometer (PerkinElmer, Santa Clara, CA, USA) calibrated with polystyrene film. Matrix-assisted laser desorption ionization time-of-flight (MALDI-TOF) mass spectra were collected on a Bruker ultrafleXtreme in the mass spectrometry laboratory, University of Illinois, with α-cyano-4-hydroxycinnamic acid (CHCA) as the matrix.

Polymerization kinetics

In a typical measurement of polymerization kinetics, BLG-NCA (52.6 mg, 0.2 mmol) were dissolved in anhydrous deuterated DCM (400 μL), into which the deuterated DCM stock solution of *n*-hexylamine (20 mM, 100 μL, $[M]_0/[I]_0 = 100$, $[M]_0 = 0.4$ M) was added to start the polymerization. The mixture was transferred into an NMR tube, and the NMR spectrum was monitored at different time intervals. In order to calculate the conversion of NCA at different time, the integral of ring N–H signal of BLG-NCA (δ = 6.4-7.1 ppm, shifting towards higher field as polymerization proceeds) was normalized compared to the ring N–H signal at $t = 0$ (*i.e.*, 100% remaining NCA). The ring N-H signal at $t = 0$ was calculated based on the integral ratios of side-chain benzyl peaks between BLG-NCA (δ = 5.13 ppm) and resulting poly(γ-benzyl-L-glutamate) (PBLG) (δ = 5.08 ppm).

Polymerization in other solvents and polymerization with other monomers were conducted in a similar way. Polymerization in DMF was carried out in a similar way, but using regular DMF as the solvent. The signals of DMF was pre-saturated (δ = 2.9, 3.1, and 8.2 ppm) in order to better reveal the signals from BLG-NCA and PBLG.

After complete conversion of NCAs, the resulting polymers were purified by precipitation in hexane:ether (1:1, v/v) and dried under vacuum. The obtained polypeptides were dissolved in DMF containing 0.1 M LiBr, filtered through a 0.45 μm PTFE membrane (Thermo Fisher Scientific, Waltham, MA, USA), and analyzed by GPC.

Saturation-transfer difference (STD) NMR studies

In order to differentiate the NMR signals from polypeptides and NCAs, ELG-NCA and PBLG with different side chains were used to probe the binding between polypeptides and NCAs in STD-NMR experiments.

PBLG with an active terminal amino group (PBLG-NH_2) was prepared by polymerization of BLG-NCA in DMF. The designed degree of polymerization (DP) was 30 to ensure an α-helical conformation. The synthesis was carried out at 4 ^{o}C to minimize the loss of amino groups at *N*-terminus through amidation reaction with side-chain benzyl esters[4]. After polymerization, PBLG-NH_2 was purified by precipitation in cold hexane:ether (1:1, v/v) and stored at -30 ^{o}C. The control polypeptide with actyl-capped *N*-terminus (PBLG-NHAc) was synthesized by reacting PBLG-NH_2 with acetic anhydride (40 equiv.) at room temperature in DMF overnight. PBLG-NHAc was then purified by precipitation in cold ether. The fidelity of *N*-terminus of PBLG-NH_2 and PBLG-NHAc was confirmed by MALDI-TOF analysis (Supplementary Fig. 7).

In a typical STD NMR experiment, the final concentration of ELG-NCA is 0.05 M to ensure slow NCA consumption by PBLG-NH_2 during NMR tests. The analysis of ring N–H signal from ELG-NCA after STD scan confirmed slow polymerization, where only < 2% NCA was consumed for each experiment (~ 30 min).

Saturation of the aromatic signal from PBLG-NH_2 or PBLG-NHAc was achieved by a train of Gaussian-shaped pulse at 7.27 ppm (aromatic peak from PBLG side chain, 1701 Hz), with a saturation power of 12 dB (B1 strength of 45 Hz) for a saturation time of 0.5, 1, 2, 3, and 4 s. A relaxation delay of 5 s was applied for each experiment. The off-resonance irradiation was set at 30 ppm (18,735 Hz) with no signals for either polypeptide or NCA.

The STD NMR in deuterated DMF was carried out in a similar way, but with an on-resonance saturation frequency at 7.52 ppm (aromatic peak from PBLG side chain, 1,888 Hz). The STD NMR for PELG and BDG-NCA was carried out in a similar way, but with an on-resonance saturation frequency at 1.23 ppm (methyl peak from PELG side chain, -2,821 Hz), and only at saturation time of 3 s. The STD NMR for PBLG and EG_2-Lys-NCA was carried out in a similar way, but with an on-resonance saturation frequency at 5.04 ppm (benzyl peak from PBLG side chain, 28 Hz), and only at saturation time of 3 s.

The STD amplification factor (A_{STD}) was calculated using the following equation:

$$A_{STD} = \frac{I_{STD}}{I_0} \times \frac{[\text{ELG-NCA}]}{[\text{PBLG}]} \quad (1)$$

Where I_{STD} and I_0 represents the signal intensities of ring N-H proton of ELG-NCA in the STD spectrum and original spectrum (*i.e.*, off-resonance spectrum), respectively.

Polymerization in the presence of water

BLG-NCA (52.6 mg, 0.2 mmol) were dissolved in anhydrous solvent (DCM or DMF, 400 μL), into which water (50 μL) was added. While water formed a separate layer on top of DCM solution, it was completely dissolved in DMF solution. The stock solution of 1-pyrenemethyl amine (20 mM, 100 μL, $[M]_0/[I]_0 = 100$, $[M]_0 = 0.4$ M) was then added into the mixture to start the polymerization. As the polymerization proceeds, the mixture turns turbid in both systems. The resulting polymers were purified by precipitation in hexane/ether (1:1, v/v), and the residual water was carefully removed by a pipette. The obtained polypeptides were dried under vacuum and analyzed by GPC.

Simulation Methods

The computational assay consisted of $PBLG_{15}$ organized in an α-helical conformation and one BLG-NCA immersed in a cubic cell containing 1,500 DCM molecules. The length of the cell at thermodynamic equilibrium was approximately equal to 56 Å, corresponding to a computational assay of 7,989 atoms.

A Langevin thermostat with a damping coefficient of 1 ps^{-1} maintained the temperature at 296 K. The Langevin piston method was used to maintain the system at a nominal pressure of 1 atm [5]. Covalent bonds involving hydrogen atoms were constrained to their equilibrium length by the Rattle algorithm [6]. Long-range electrostatic forces were evaluated using the particle-mesh Ewald algorithm [7] with a grid spacing of 1.2 Å, while a smoothed 12-Å spherical cutoff was applied to truncate short-range van der Waals and electrostatic interactions. The r-RESPA multiple time-stepping algorithm [8] was employed to integrate the equations of motion with an effective time step of 2×10^{-15} s for short-range interaction and 4×10^{-15} s long-range interactions.

To probe the binding interactions between an NCA and a coiled $PBLG_5$, one hundred independent 10-ns simulations were spawned with distinct seeds after suitable thermalization, corresponding to an aggregate simulation time of 1 μs.

The transition coordinate r was defined as the Euclidian distance separating the center of mass of the first three $-NH_2$ or amide N-H of $PBLG_{15}$ from that of the three oxygen atoms of the NCA ring moiety. The reaction pathway spanning 16 Å; *i.e.*, $2 \leq r \leq 18$ Å, was discretized in bins 0.1 Å wide, in which samples of the local force acting along r were accumulated. To enhance ergodic sampling, we turned to a multiple-walker [9] version of the eABF algorithm, using four independent walkers, from whence the variance on the free energy was inferred. The aggregate simulation time amounted to 6×10^{-6} s.

Integration of the PMF yields the association constant [10],

$$K_a = 4\pi \int_0^{r^*} \mathrm{d}r \; r^2 e^{-\beta\omega(r)} \qquad (2)$$

$\beta = 1/k_B T$, where k_B is the Boltzmann constant and T is the temperature. r^* is the limit of interaction, chosen here equal to 12 Å. The standard binding free energy can be recovered as $\Delta G° = -1/\beta \ln(K_a C°)$, where $C°$ is the standard concentration.

Kinetic Modeling

Based on the kinetic model:

$$M_i^* + M \xrightarrow{k_1} M_{i+1}^* \qquad 1 \leq i < s \qquad (3)$$

$$M_i^* + M \underset{k_{off}}{\overset{k_{on}}{\rightleftarrows}} M_i^* - M \xrightarrow{k_r} M_{i+1}^* \qquad i \geq s \qquad (4)$$

It is straightforward to write down the kinetic equations, with $[X]$ representing the concentrations of various species:

$$\frac{\partial [M_1^*]}{\partial t} = -k_1 [M_1^*][M] \qquad i = 1 \qquad (5)$$

$$\frac{\partial [M_i^*]}{\partial t} = k_1 [M]([M_{i-1}^*] - [M_i^*]) \qquad 1 < i < s \qquad (6)$$

$$\frac{\partial [M_s^*]}{\partial t} = k_1 [M][M_{s-1}^*] - k_{on} [M_s^*][M] + k_{off} [M_s^* - M] \qquad i = s \qquad (7)$$

$$\frac{\partial [M_i^*]}{\partial t} = -k_{on} [M_i^*][M] + k_{off} [M_i^* - M] + k_r [M_{i-1}^* - M] \qquad i > s \qquad (8)$$

$$\frac{\partial [M_i^* - M]}{\partial t} = k_{on}[M_i^*][M] - k_{off}[M_i^* - M] - k_r[M_i^* - M] \qquad i \geq s \tag{9}$$

$$FM1 = \sum_{i=s}^{\infty}[M_i^*] \qquad FM2 = \sum_{i=s}^{\infty}[M_i^* - M]$$

$$\frac{\partial FM1}{\partial t} = (k_r + k_{off})FM2 - k_{on}[M]FM1 + k_1[M][M_{s-1}^*] \tag{10}$$

$$\frac{\partial FM2}{\partial t} = -(k_r + k_{off})FM2 + k_{on}[M]FM1 \tag{11}$$

$$\frac{\partial [M]}{\partial t} = -k_1[M]\sum_{i=1}^{s-1}[M_i^*] - k_{on}[M]FM1 + k_{off}FM2 \tag{12}$$

Additionally, by defining:

$$\tau = tk_1M_0,\ x = \frac{[M]}{[M_0]},\ x_i = \frac{[M_i^*]}{[M_0]},\ y_i = \frac{[M_i^* - M]}{[M_0]},\ f_1 = \frac{FM1}{[M_0]},\ f_2 = \frac{FM2}{[M_0]}$$

The kinetic model can be written as:

$$x_i + x \xrightarrow{k_1} x_{i+1} \qquad 1 \leq i < s \tag{13}$$

$$x_i + x \underset{k_{off}}{\overset{k_{on}}{\rightleftarrows}} y_i \xrightarrow{k_r} x_{i+1} \qquad i \geq s \tag{14}$$

We can then write the kinetic equations into dimensionless form:

$$\frac{\partial [x_1]}{\partial \tau} = -[x_1][x] \qquad i = 1 \tag{15}$$

$$\frac{\partial [x_i]}{\partial \tau} = [x]([x_{i-1}] - [x_i]) \qquad 1 < i < s \tag{16}$$

$$\frac{\partial [x_s]}{\partial \tau} = [x][x_{s-1}] - \frac{k_{on}}{k_1}[x_s][x] + \frac{k_{off}}{k_1}[y_s]/[M_0] \qquad i = s \tag{17}$$

$$\frac{\partial [x_i]}{\partial \tau} = -\frac{k_{on}}{k_1}[x_i][x] + \frac{k_{off}}{k_1}[y_i]/[M_0] + \frac{k_r}{k_1}[y_{i-1}]/[M_0] \qquad i > s \tag{18}$$

$$\frac{\partial [y_i]}{\partial \tau} = \frac{k_{on}}{k_1}[x_i][x] - \frac{(k_{off} + k_r)}{k_1}[y_i]/[M_0] \qquad i \geq s \tag{19}$$

$$f_1 = \sum_{i=s}^{\infty}[x_i] \qquad f_2 = \sum_{i=s}^{\infty}[y_i]$$

$$\frac{\partial f_1}{\partial \tau} = \frac{(k_r + k_{off})}{k_1}f_2/[M_0] - \frac{k_{on}}{k_1}[x]f_1 + [x][x_{s-1}] \tag{20}$$

$$\frac{\partial f_2}{\partial \tau} = -\frac{(k_r + k_{off})}{k_1} f_2 / [M_0] + \frac{k_{on}}{k_1}[x] f_1 \tag{21}$$

$$\frac{\partial [x]}{\partial \tau} = -[x]\sum_{i=1}^{s-1}[x_i] - \frac{k_{on}}{k_1}[x] f_1 + \frac{k_{off}}{k_1} f_2 / [M_0] \tag{22}$$

The initiation step was omitted by assuming that all initiators were initiated at the first data point (t = 2.5 or 3.5 min). To get unique fitting results for the rate constants, three replicates with slightly different monomer concentrations for each of the nine conditions were fitted with exactly the same parameters.

Supplementary Figures

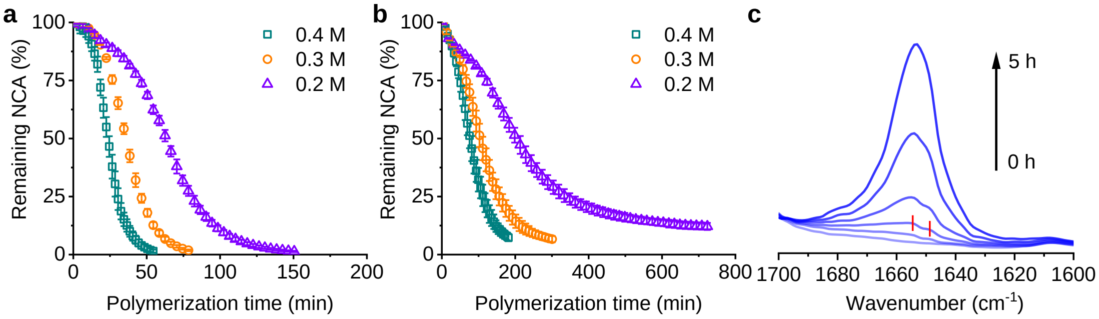

Supplementary Figure 1. Polymerization of BLG-NCA in DCM. **a**, **b** Conversion of BLG-NCA initiated by *n*-hexylamine in anhydrous DCM at various $[M]_0$ with $[M]_0/[I]_0 = 50$ (**a**) and 150 (**b**). Error bars represent standard deviations from three independent measurements. **c** FTIR spectra monitoring the polymerization of BLG-NCA in DCM ($[M]_0/[I]_0 = 100$, $[M]_0 = 0.05$ M). The highlighted peaks are 1649 and 1655 cm^{-1} that suggesting the formation of α-helical and random-coiled conformations (amide I) at the early stages of the polymerization.

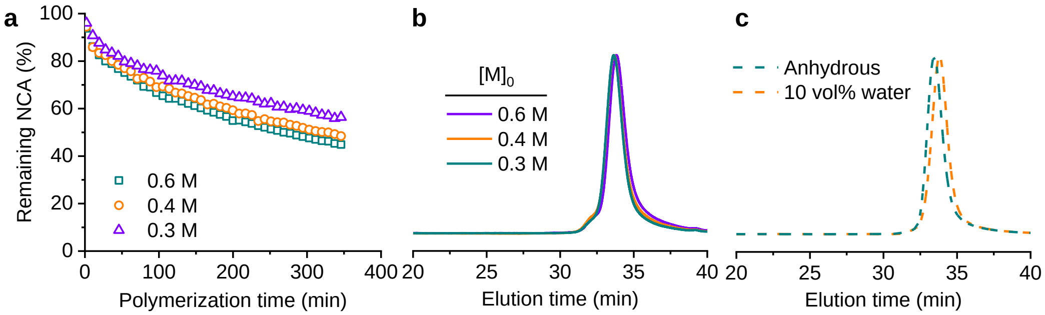

Supplementary Figure 2. Polymerization of BLG-NCA in DMF. **a** Conversion of BLG-NCA initiated by *n*-hexylamine in anhydrous DMF at various $[M]_0$ with $[M]_0/[I]_0$ = 100. **b** Normalized GPC-LS traces of obtained PBLG from polymerization in anhydrous DMF. The polymerization results are summarized in Supplementary Table 3. **c** Normalized GPC-LS traces of obtained PBLG from polymerization of BLG-NCA in DMF under anhydrous conditions or in the presence of 10 vol% water. $[M]_0$ =0.4 M, $[M]_0/[I]_0$ = 100.

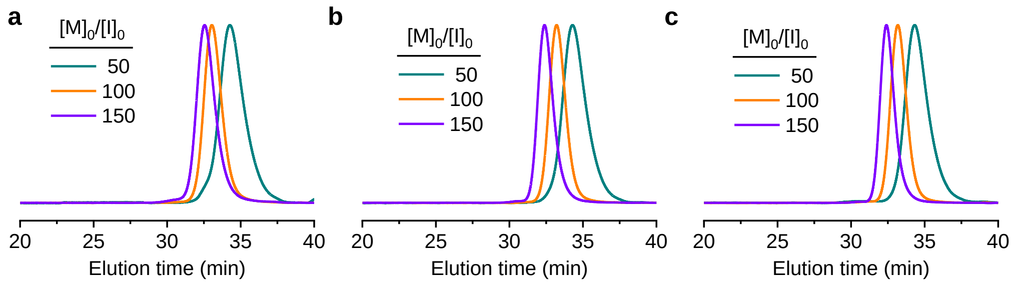

Supplementary Figure 3. Normalized GPC-LS traces of obtained PBLG from polymerization of BLG-NCA initiated by *n*-hexylamine in anhydrous DCM with various $[M]_0/[I]_0$ ratios at $[M]_0$ = 0.2 M (**a**), 0.3 M (**b**), and 0.4 M (**c**).

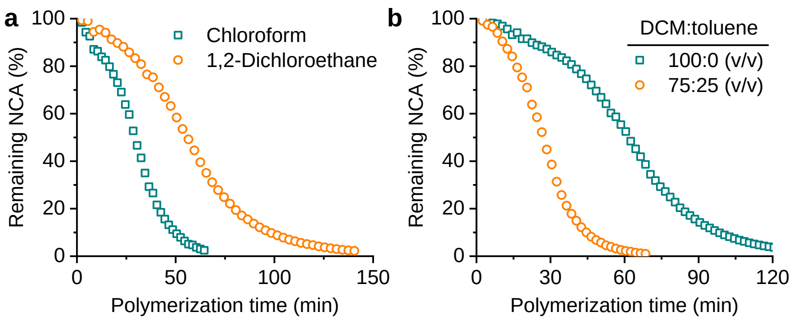

Supplementary Figure 4. Polymerization of BLG-NCA in other non-polar solvents. **a** Conversion of BLG-NCA initiated by *n*-hexylamine in anhydrous chloroform (ε = 4.81) and 1,2-dichloroethane (ε = 10.65). **b** Conversion of BLG-NCA initiated by *n*-hexylamine in anhydrous DCM in the absence and in the presence of 25 vol% of toluene (ε = 2.38). $[M]_0/[I]_0 = 50$, $[M]_0 =$ 0.2 M.

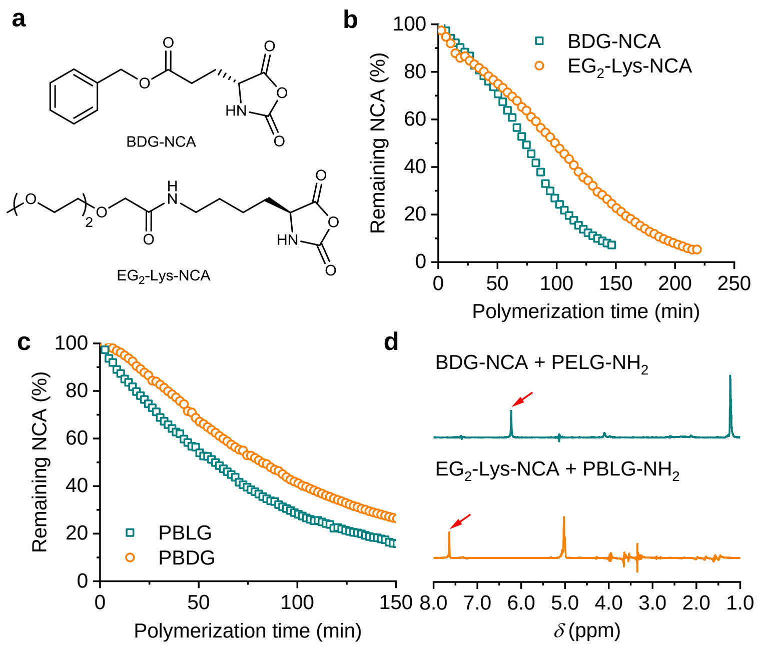

Supplementary Figure 5. Self-catalyzed polymerization of other NCAs bearing different chirality and side-chain structures in DCM. **a** Chemical structures of BDG-NCA and EG_2-Lys-NCA. **b** Conversion of BDG-NCA and EG_2-Lys-NCA in DCM. $[M]_0/[I]_0 = 50$, $[M]_0 = 0.2$ M. **c** Polymerization of BLG-NCA in DCM with PBLG or PBDG macroinitiators. $[M]_0/[I]_0 = 100$, $[M]_0 = 0.4$ M. **d** STD NMR spectra of BDG-NCA in the presence of PELG-NH_2 (top) and EG_2-Lys-NCA in the presence of PBLG-NH_2. The STD signals of ring N-H proton from NCAs were highlighted with red arrows. The peak at 1.23 ppm (top) and 5.04 ppm (bottom) are the irradiation peaks since no background suppression was applied.

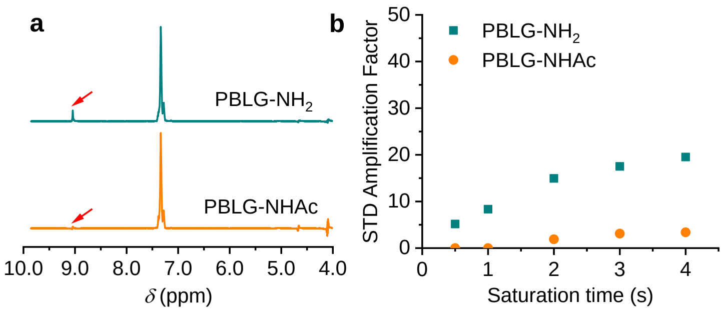

Supplementary Figure 6. STD NMR results in DMF. **a** STD NMR spectra of ELG-NCA in the presence of PBLG-NH_2 or PBLG-NHAc. The STD signal of ring N-H proton was highlighted with red arrows. The peak at 7.52 ppm is the irradiation peak since no background suppression was applied. **b** STD amplification factor of ring N-H proton of ELG-NCA in the presence of PBLG-NH_2 or PBLG-NHAc at various saturation times.

Compared with STD signals in DCM, the signals in DMF are much smaller in the presence of both PBLG-NH_2 and PBLG-NHAc, which indicates weaker interactions between *N*-terminus of polypeptides and NCA monomers.

a

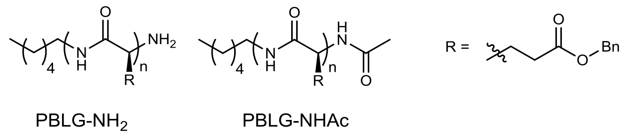

b

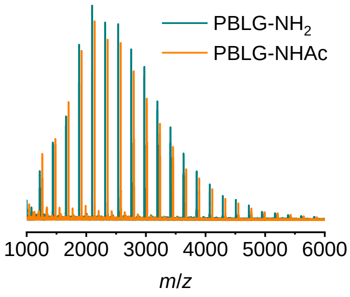

c

n	PBLG-NH_2		PBLG-NHAc	
	Calcd	Found	Calcd	Found
8	1876.8	1877.0	1918.8	1919.1
9	2095.9	2096.0	2137.9	2138.2
10	2315.0	2315.1	2357.0	2357.3
11	2534.1	2534.2	2576.1	2576.4
12	2753.2	2753.3	2795.2	2795.5
13	2972.3	2972.4	3014.3	3014.6

Supplementary Figure 7. MALDI-TOF characterization of polypeptides used in STD NMR. **a** Chemical structures of PBLG-NH_2 and PBLG-NHAc. **b** Overlaid MALDI-TOF spectra of PBLG-NH_2 and PBLG-NHAc. **c** Comparison of representative *m*/*z* signals between calculated values from molecular formula and obtained values from MALDI-TOF spectrum.

The obtained *m*/*z* signals for PBLG-NH_2 agree well with the calculated values (124.11 + 219.09*n*) ($[M+Na]^+$), indicating negligible degradation of terminal amino groups.

The obtained *m*/*z* signals for PBLG-NHAc are consistent with the calculated values (166.12 + 219.09*n*) ($[M+Na]^+$). The shift of molecular weight ($\Delta m/z = 42$) corresponds to the added acetyl group and confirms the end-capping of *N*-terminus.

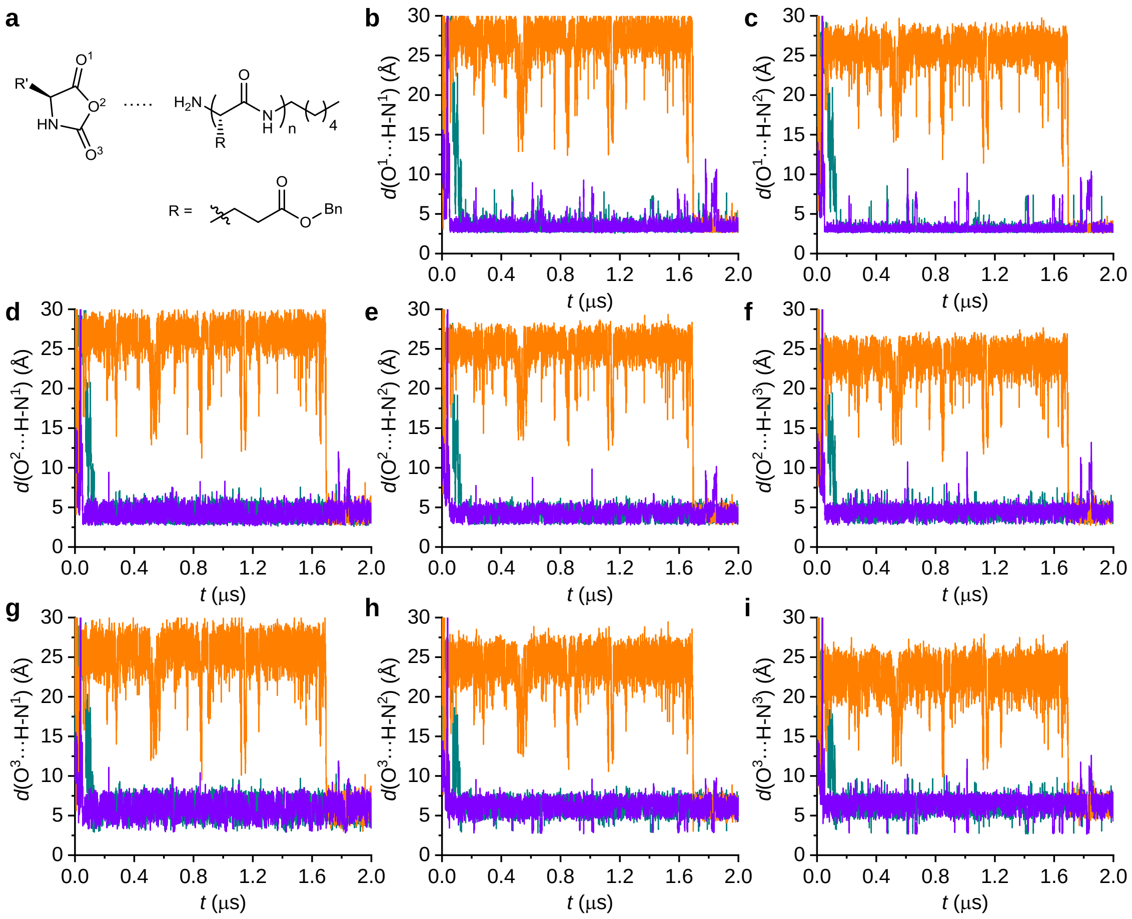

Supplementary Figure 8. H-bond analysis of polypeptide/NCA binding. **a** Chemical structures of BLG-NCA and PBLG-NH_2. **b-i** Time evolution of H-bonds formed between oxygen atoms of BLG-NCA and the amino or amide N-H groups of PBLG-NH_2. Each line represents one independent, 2-μs long MD simulation.

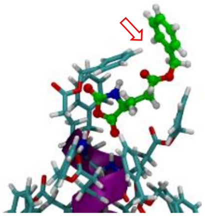

Supplementary Figure 9. Closeup from the simulation trajectory at the *N*-terminus of PBLG-NH_2 to reveal the T-shaped π-π interaction (highlighted with the red arrow).

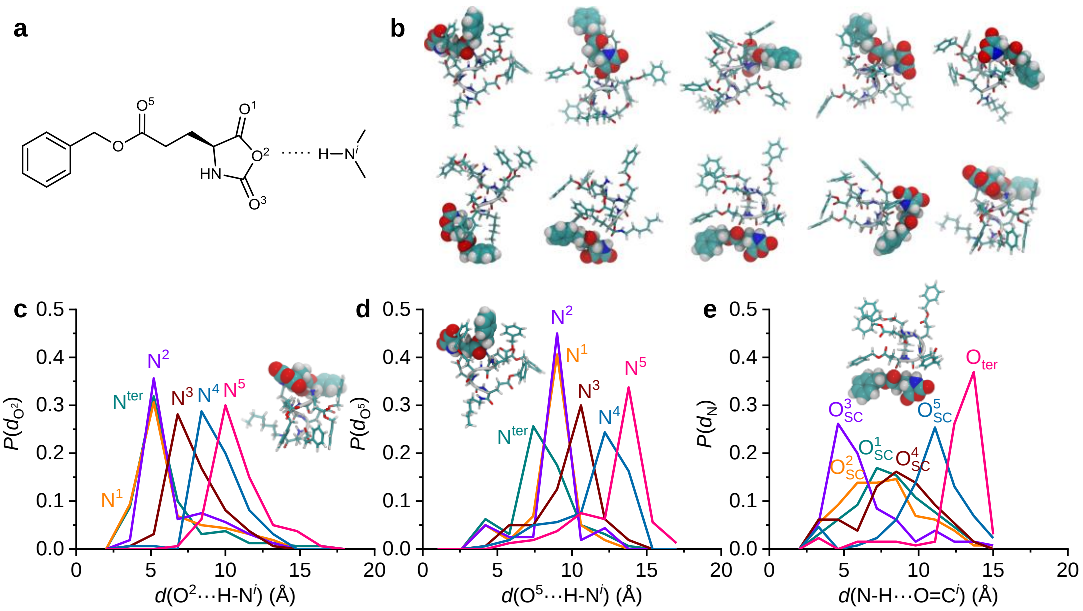

Supplementary Figure 10. Analysis of the simulations on binding interactions of the NCA and a coiled $PBLG_5$. **a** Atom labeling of BLG-NCA. **b** Snapshots of representative binding motifs and interactions of an NCA and a coiled $PBLG_5$. **c-e** Probability distributions of forming H-bonds between coiled $PBLG_5$ and one NCA. **c** The O^2 atom of NCA and the N-terminus (N^{ter}) or amide groups of $PBLG_5$. **d** The O^5 atom of NCA and the N-terminus (N^{ter}) or amide groups of $PBLG_5$. **e** The N atom of NCA and the C-terminus (O_{ter}) or side-chain ester groups of $PBLG_5$.

We have considered a computational assay consisting of a pentapeptide immersed in DCM, in the presence of one BLG-NCA. $PBLG_5$ is organized in a coil. Analysis of the trajectories reveals that in 98 out of 100 simulations association of the NCA with $PBLG_5$ was observed. Aside from the desired binding interaction with the *N*-terminus, several alternate binding poses are also monitored. In particular, through its ester moiety, the NCA can form H-bonds predominantly with the N-terminus of $PBLG_5$. Furthermore, through its N-H group, the NCA can also act as a hydrogen-bond donor, interacting primarily with the ester moiety of the pentapeptide side chains and the amide group of the *C*-terminus blocking group. As can be seen from Fig. b, irrespective of the donor and the acceptor, hydrogen-bond formation is generally accompanied by ancillary interactions, *e.g.*, T-shaped and stacking π-π interactions, stabilizing the supramolecular assembly.

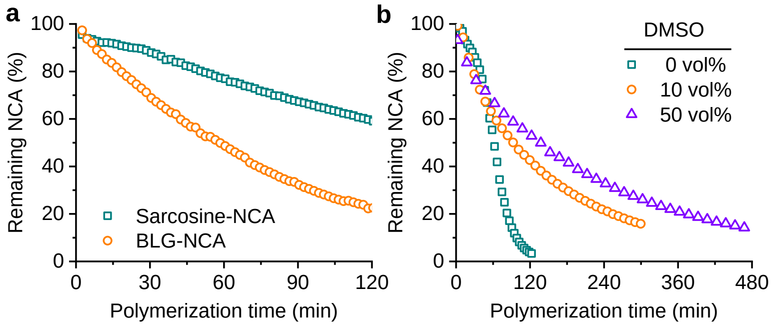

Supplementary Figure 11. Control polymerization with disrupted H-bonding interactions. **a** Conversion of Sarcosine-NCA and BLG-NCA initiated by PBLG macroinitiators in DCM. $[M]_0/[I]_0$ = 100, $[M]_0$ = 0.4 M. **b** Conversion of BLG-NCA initiated by *n*-hexylamine in DCM in the presence of different contents of DMSO as hydrogen bonding disruptors. $[M]_0/[I]_0$ = 100, $[M]_0$ = 0.4 M.

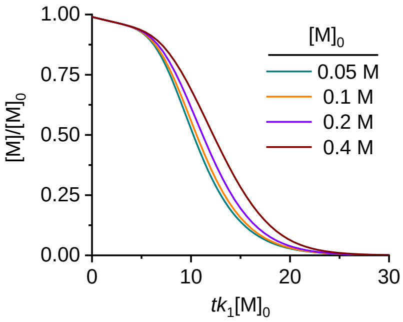

Supplementary Figure 12. Plots of the fraction of monomer versus rescaled time ($\tau = tk_1M_0$) for test cases with s = 10, $[M]_0/[I]_0$ = 100, k_1 = 0.02 $M^{-1}s^{-1}$, k_{on} = 10 $M^{-1}s^{-1}$, k_{off} = 2 s^{-1}, k_r = 0.2 s^{-1}, and at selected values of $[M]_0$ = 0.05, 0.1, 0.2, or 0.4 M.

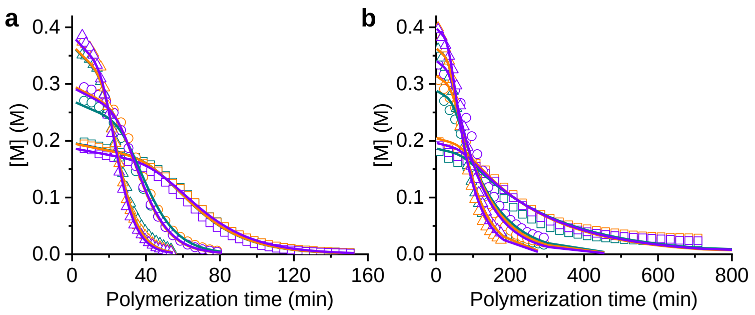

Supplementary Figure 13. Polymerization kinetics in Supplementary Fig. 1 were fit with the adsorption-incorporated kinetic model. $[M]_0/[I]_0$ = 50 (**a**) and 150 (**b**).

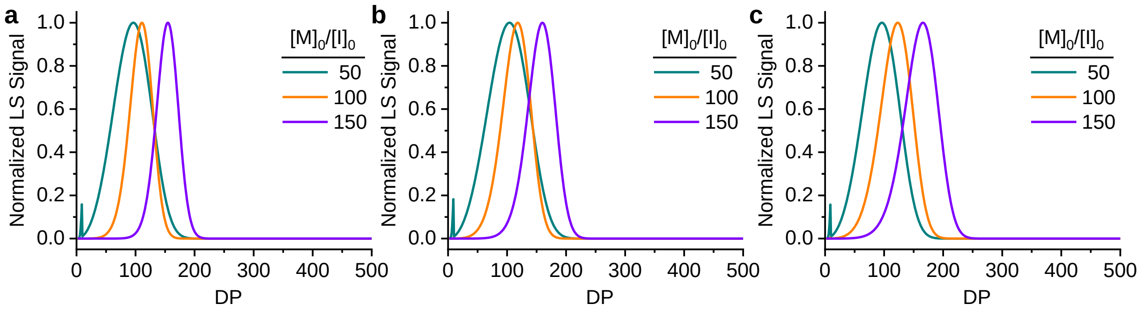

Supplementary Figure 14. Predicted GPC-LS traces with various $[M]_0/[I]_0$ ratios at $[M]_0$ = 0.2 M (**a**), 0.3 M (**b**), and 0.4 M (**c**).

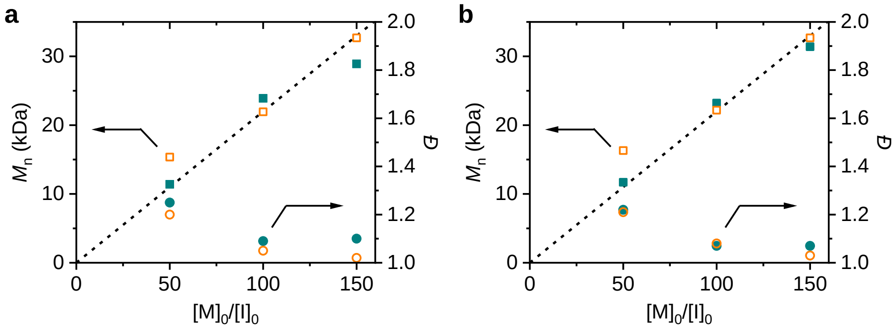

Supplementary Figure 15. Comparison of experimental results obtained from GPC analysis (solid symbols) and predicted results from modeling (open symbols) with various $[M]_0/[I]_0$ ratios at $[M]_0$ = 0.2 M (**a**) and 0.3 M (**b**). The dashed line represents designed MW by $[M]_0/[I]_0$ ratio.

Supplementary Tables

Supplementary Table 1. Characterization of resulting polypeptides from polymerization of BLG-NCAs in the presence of water.[a]

Solvent	Water (vol%)	M_n/M_n* [b,c] (kDa)	$Đ$[c]
DCM	0	25.0/22.0	1.05
	10	24.9/22.0	1.05
DMF	0	23.3/22.0	1.05
	10	20.4/22.0	1.05

[a] All polymerizations were conducted at room temperature initiated by 1-pyrenemethyl amine. [b] Obtained MW/designed MW*. [c] Determined by GPC.

Supplementary Table 2. Rate constants obtained by fitting the polymerization kinetics with the two-stage kinetic modeling incorporating an adsorption step.[a]

$[M]_0$ (M)	$[M]_0/[I]_0$	k_1 (M^{-1} s^{-1})	k_{on}/k_{off} (M^{-1})
0.2	50	0.015	0.54
	100	0.011	0.15
	150	0.011	0.11
0.3	50	0.017	0.77
	100	0.012	0.23
	150	0.011	0.18
0.4	50	0.021	0.89
	100	0.014	0.33
	150	0.011	0.24

[a] Polymerization was conducted in anhydrous DCM at room temperature initiated by *n*-hexylamine. Rate constants were determined from best fits of the kinetic model.

Supplementary Table 3. Characterization of resulting polypeptides from control polymerization of BLG-NCAs in DMF.[a]

$[M]_0$ (M)	$[M]_0/[I]_0$	M_n/M_n* [b,c] (kDa)	$Đ$[c]
0.3	100	21.7/22.0	1.05
0.4	100	23.0/22.0	1.05
0.6	100	22.9/22.0	1.05

[a] All polymerizations were conducted in anhydrous DMF at room temperature initiated by *n*-hexylamine. [b] Obtained MW/designed MW*. [c] Determined by GPC.

Supplementary References

1. Baumgartner, R., Fu, H., Song, Z., Lin, Y. & Cheng, J. Cooperative polymerization of α-helices induced by macromolecular architecture. *Nat. Chem.* **9**, 614-622 (2017).
2. Yu, M., Nowak, A. P., Deming, T. J. & Pochan, D. J. Methylated mono- and diethyleneglycol functionalized polylysines: nonionic, α-helical, water-soluble polypeptides. *J. Am. Chem. Soc.* **121**, 12210-12211 (1999).
3. Fetsch, C., Grossmann, A., Holz, L., Nawroth, J. F. & Luxenhofer, R. Polypeptoids from *N*-substituted glycine *N*-carboxyanhydrides: hydrophilic, hydrophobic, and amphiphilic polymers with poisson distribution. *Macromolecules* **44**, 6746-6758 (2011).
4. Habraken, G. J. M., Peeters, M., Dietz, C. H. J. T., Koning, C. E. & Heise, A. How controlled and versatile is *N*-carboxy anhydride (NCA) polymerization at 0 ℃? Effect of temperature on homo-, block- and graft (co)polymerization. *Polym. Chem.* **1**, 514-524 (2010).
5. Feller, S. E., Zhang, Y., Pastor, R. W. & Brooks, B. R. Constant pressure molecular dynamics simulation: the Langevin piston method. *J. Chem. Phys.* **103**, 4613-4621 (1995).
6. Andersen, H. C. Rattle - a "velocity" version of the shake algorithm for molecular dynamics calculations. *J. Comput. Phys.* **52**, 24-34 (1983).
7. Darden, T., York, D. & Pedersen, L. Particle mesh Ewald - An *N* log(*N*) method for Ewald sums in large systems. *J. Chem. Phys.* **98**, 10089-10092 (1993).
8. Tuckerman, M., Berne, B. J. & Martyna, G. J. Reversible multiple time scale molecular dynamics. *J. Chem. Phys.* **97**, 1990-2001 (1992).
9. Comer, J., Phillips, J. C., Schulten, K. & Chipot, C. Multiple-replica strategies for free-energy calculations in NAMD: multiple-walker adaptive biasing force and walker selection rules. *J. Chem. Theory Comput.* **10**, 5276-5285 (2014).
10. Shoup, D. & Szabo, A. Role of diffusion in ligand-binding to macromolecules and cell-bound receptors. *Biophys. J.* **40**, 33-39 (1982).
