# Supplementary material for: Enzyme-mimetic self-catalyzed polymerization of polypeptide helices
Source: Nat Commun. 2019 Nov 29;10:5470. doi: 10.1038/s41467-019-13502-w (PMC6884638; doi:10.1038/s41467-019-13502-w)
Supplement: Supplementary file 2 — Description of Additional Supplementary Files [file 41467_2019_13502_MOESM2_ESM.docx]

Description of Additional Supplementary Files

**Supplementary Movie 1 and 2:** Movies illustrating the binding between PBLG15-NH2 and BLG-NCA in DCM. The movies are excerpts of two independent, 2-μs long simulations. The solvent has been omitted for clarity.
